# Weaving the Digital Tapestry: Methods for Emulating Cohorts of Cardiac Digital Twins Using Gaussian Processes

**DOI:** 10.1007/s10439-025-03890-0

**Published:** 2025-11-17

**Authors:** Christopher W Lanyon, Cristobal Rodero, Abdul Qayyum, Tiffany MG Baptiste, Steven A Niederer, Richard D Wilkinson

**Affiliations:** 1 School of Mathematical Sciences, University of Nottingham, Nottingham, UK; 2 Cardiac Electro-Mechanics Research Group (CEMRG), National Heart and Lung Institute, Faculty of Medicine, Imperial College London, London, UK; 3 School of Biomedical Engineering and Imaging Science, Kings College London, London, UK; 4 Turing Research and Innovation Cluster in Digital Twins (TRIC: DT), The Alan Turing Institute, London, UK

**Keywords:** Digital twins, Gaussian Processes, Cohort Learning, Machine Learning, Cardiac Modelling

## Abstract

**Purpose:**

Digital twin (DT) cohorts are collections of models where each member represents an individual real-world asset. DT cohorts can be used for in-silico trials, outlier detection and forecasting, and are used across engineering, industry, and increasingly in personalised medicine. To increase the scalability of DT cohorts, researchers often train emulators to be used as cheap surrogates of computationally expensive mathematical models. Frequently, each cohort member is emulated individually, without reference to other members. We propose that instead, we can treat each DT as a thread in a larger network, and that these threads can be woven together into a digital tapestry using cohort learning methods.

**Methods:**

We propose two statistical approaches for transferring knowledge between threads. The first method, ‘latent-feature emulators’, utilises a latent representation of individual cohort members to generate a single emulator for the entire cohort. The second method, ‘discrepancy emulators’, learns the discrepancy between a new cohort member and existing members.

**Results:**

In two cardiac DT case studies, we show that these methods can reduce computational costs by more than 50% compared to the standard approach of training individual emulators, even in small cohorts.

**Conclusions:**

We find that by transferring information between meshes, the cohort methods improve both the computational efficiency and the accuracy of emulators when compared to the standard approach of individually emulating each cohort member. As cohort size increases, the computational savings grow further. We focus on the use of Gaussian process emulators, but the transfer methods are applicable to other surrogate approaches such as neural networks.

**Supplementary Information:**

The online version contains supplementary material available at 10.1007/s10439-025-03890-0.

## Introduction

Digital twins (DTs) are mathematical or computer models tailored to specific instances of real-world systems or processes [1–6]. DTs are increasingly used in healthcare [7], engineering [8], and manufacturing and industry [9] to provide personalised recommendations, prognosis, and future predictions. DTs are often built around complex multi-scale mathematical models that are expensive and slow to run, meaning it can be hard to utilise them in fast-paced real-world settings, such as clinical environments [3]. In some scenarios, researchers may seek to generate a cohort of DTs, for example, a cohort of heart patients, each with their own personalised cardiac DT. This provides an opportunity to share information across the cohort, to reduce the cost of each new DT.

Computer model emulation or surrogate modelling is widely used to mitigate the computational cost of DTs [8, 10–13]. A statistical or machine learning model (such as a Gaussian process [14] or neural network [15]) is used to approximate the input-output relationship of the computer model, trained using an ensemble of model evaluations for some design of different input variables. The simplest approach for cohorts is to train an independent emulator for each cohort member, requiring us to create a new ensemble of simulations for each case. For example, for the cardiac DTs considered later, each patient has a unique cardiac geometry, requiring a unique set of simulations tailored to their anatomy. We propose an alternative approach, where each new DT added to the cohort learns from existing cohort members by exploiting the similarities across the individual models, weaving together individual DT threads into what we call a *digital tapestry*.

We propose and test two methods to form this digital tapestry: Firstly, a single Gaussian process emulator (GPE) that learns using latent variables that distinguish the members of the cohort. Secondly, a *discrepancy emulator* that inherits the mean and covariance functions from a cohort of existing emulators and learns the discrepancy between the cohort and the new member. This discrepancy emulator method also allows the sequential addition of new cohort members whilst still utilising a meta-learning approach, distinguishing it from other methods of DT emulation.

We test the approaches on two case studies used in the study and treatment of cardiac diseases. Firstly, a cohort of cardiac DTs [11] where each DT is comprised of a patient-specific full-heart geometry, represented as a computational mesh over which the reaction-eikonal model for electrophysiology (EP) can be simulated. Secondly, a cohort of atrial DTs used to model the mechanical function of the left atrium in patient-specific atrial geometries. We find that using cohort learning to transfer information between the anatomical models we can outperform individual emulators at a significantly reduced computational cost.

### Related Work

Emulators, also known as surrogates or metamodels are used widely in many disciplines, including cardiac modelling [11, 12, 16–21], personalised medicine [22–24], structural health monitoring [25–27], groundwater modelling [28], dynamical systems [10], and glaciology [13, 29, 30]. They enable tasks that would usually require many simulator evaluations, such as global sensitivity analysis (GSA) [12], history matching [31, 32] and model calibration [33], to be achieved at reasonable computational cost. For concreteness, we focus on Gaussian process emulators, as they are well-established in the literature [34] and possess desirable statistical properties [14], but the structural assumptions we introduce for transfer learning across the cohort are not emulator specific (i.e., they can equally be used in the context of neural networks, low rank tensor-trains, etc).

Many other authors have looked at methods to learn about or from populations of models, and here we give a brief non-exhaustive overview of how our work relates to prior work. What we call *latent-feature* emulators are similar to population methods in structural health modelling (SHM). For example, in [35] a Gaussian mixture model is used as a DT for a cohort of wind turbines in a wind farm. They treat the population of turbines as homogeneous, as very little variation is expected between turbines, allowing the entire cohort to be modelled with a single emulator, enabling identification of population outliers (such as damaged turbines). They extend this population-based approach to SHM for heterogeneous structures [36–38], establishing the conditions under which structures can be considered similar enough for transfer learning to be applicable, and then use graph neural networks to achieve transfer learning by projecting individual structures onto points in abstract space, in order to compare non-identical structures and assess structure similarity. In our work, rather than using a graph representation of the different cohort members, we use kernel methods to define the space of possible functions and then learn latent features to specify the particular function in any given case. But our aim is similar: we want to generate a model that can represent the entire population, and which can thus transfer learning between samples.

The second method we propose, the discrepancy emulator*,* learns to approximate the simulation of a new cohort member by incorporating information from all previous cohort members. This draws parallels with ensemble learning, which is a group of methods that combine the outputs of several predictive models, e.g., as a weighted sum, to improve predictions [39]. Ensemble learning is useful in cases where the optimal solution is outside of the predictive scope of any single model, or when the user is unsure of which model is best suited to a certain scenario. For example, in climate science, ensemble modelling is used to better characterize the range of potential future climate events [40]. The discrepancy emulator method we propose combines this weighting approach with a *meta-learner* [39], which learns the discrepancy between the new cohort member and the output of the ensemble model. In our examples, this proves to be particularly effective and data efficient.

There are also similarities between our work and multi-task Gaussian processes [41], which have also been used in the context of personalised medicine [42]. Multi-task GPs are used to model sets of non-independent outputs or “tasks”. For example, in the MAGMA framework [43, 44], the time dependent output for a given task, ${y}_{i}\left(t\right),$ is modelled as

1 $${y}_{i}\left(t\right)= \mu \left(t\right)+ {f}_{i}\left(t\right)+ {\epsilon }_{i},$$

where $\mu$ is the mean behaviour of all tasks, ${f}_{i}$ is the task specific behaviour, and ${\epsilon }_{i}$ is a normally distributed error. This is essentially a non-parametric multi-level model [45]. Our discrepancy emulator takes a similar form, except that instead of having a mean behaviour which applies to all cohort members, we assume that a new cohort member can be described by a weighted sum of the previous members plus some discrepancy term.

## Materials and Methods

Consider a set of similar real-world systems, ${S}_{1},{S}_{2},\dots$ for which we wish to create digital twins. We will assume that all of the systems are different, but of the same type. For example, each system may correspond to the heart of a patient being treated for a specific cardiovascular disease, as in our case studies. The key element of each digital twin is a mathematical model, ${f}_{i}\left(\theta \right)$, where $\theta \in\Theta$ is a $k$ dimensional input parameter. As each system is of the same type, the functions ${f}_{1},{f}_{2}$,… may all be similar in some way (e.g., they all model the same cardiovascular function), but because they have each been tailored to a specific real-world system, they will not be identical (e.g. each patient is unique). For each system, ${S}_{i},$ its corresponding model, ${f}_{i}$, is comprised of a personalised mesh and a set of model equations depending on parameters $\theta$. For example, the geometry of the heart with partial differential equations defined on that geometry describing some process (such as electrophysiology). Throughout this work we assume that the underlying model equations are the same for each system. The functional differences between systems are due to patient-specific geometries (represented by the mesh) and parameters $\theta$.

In cases where the mathematical models, ${f}_{i}$, are computationally intensive, a common approach is to train an emulator of the simulator. We seek a model ${g}_{i}$ that approximates ${f}_{i}$ so that ${f}_{i}\left(\theta \right)\approx {g}_{i}\left(\theta \right)$ for all $\theta$, but which is fast to evaluate. Ideally, ${g}_{i}$ will also provide a quantification of the accuracy of the approximation, usually in the form a probability distribution. There are many approaches to building emulators, but we focus on empirical machine learning emulators that are trained using a set of simulations from each *f*_*i*_. Methods for creating an emulator of a single function are well-established [46]. Our focus here is on how we create emulators of multiple-related systems. The hope is that by moving from approximating one function at a time, to emulating entire cohorts of functions, we can find efficient computational strategies. In the language of digital twins, we say that each system has its own digital thread consisting of simulations and data. By considering multiple different threads from different systems, a *digital tapestry* can be woven, eventually allowing us to add new cohort members with minimal computational cost.

### Gaussian Process Emulators

We focus on Gaussian process (GP) models, which broadly can be considered to be probability distributions over a space of functions. A GP is fully specified by its mean, $\mu \left(\cdot \right)$, and covariance function, $\kappa \left(\cdot,\cdot \right)$:

2 $$g\left(\cdot \right)\sim GP\left(\mu \left(\cdot \right),\kappa \left(\cdot,\cdot \right)\right)$$

These functions may contain hyperparameters that need to be estimated from data, for example, by finding their maximum likelihood estimates. For details on the mean and covariance functions used in this paper see Appendix A.

We compare three methods for emulating DT cohorts: individual emulators for each system, an emulator which learns from *latent features*, and *discrepancy emulators*. The latter two methods leverage the concept of the digital tapestry to learn from the full cohort of simulations.

#### Individual Emulators for Each Cohort Member

The simplest approach is to build an independent emulator for each DT in the cohort. For each ${f}_{i},$ we build a GPE, ${g}_{i}$, learnt solely from simulations of ${f}_{i}$ without reference to information from other cohort members. This means that each cohort member will incur approximately the same computational cost, as a similar number of evaluations of ${f}_{i}$ will be required in each case. We treat these individual emulators as a baseline for our cohort learning methods.

To generate a GPE for a single DT, system *S*_*i*_ say, we first evaluate ${f}_{i}\left(\theta \right)$ over ${J}_{i}$ values of $\theta$ to create an ensemble of simulations that we use to train the emulator: ${D}_{i}={\left\{{\theta }_{j},{f}_{i}\left({\theta }_{j}\right)\right\}}_{j=1}^{{J}_{i}}$. Standard Gaussian process regression can then be used to find posterior mean and covariance functions, with hyperparameters learnt, e.g., by maximum likelihood [47]. Algorithm 1 below describes the process of creating a cohort of individual emulators. Though the methods presented here are sufficiently general to extend to multi-output GPs in cases where our computer model output, ${f}_{i}\left(\theta \right)$, is multidimensional with N dimensions, i.e., ${f}_{i}\left(\theta \right)=({f}_{i1}\left(\theta \right), \dots , {f}_{iN}\left(\theta \right))^\top$, we train independent GPEs for each output, so that ${g}_{i}\left(\theta \right)=({g}_{i1}\left(\theta \right),\dots ,{g}_{iN}\left(\theta \right))^\top$. This choice ensures that any computational savings or predictive accuracy improvements are due solely to the cohort learning methods we introduce, not the learned relationships between function outputs. The methods we propose can be generalized to the multi-output case, but given using independent emulators for each output is the most common approach in the literature [11, 12, 19], we limit ourselves to this case.

Using individual emulators for each cohort member is a natural and computationally simple approach, allowing each cohort member to be added sequentially. However, it can be computationally expensive and does not allow us to leverage knowledge gained by building many such systems, and may limit the predictive accuracy of the resulting emulators. Our hypothesis is that with a cohort learning method we can achieve similar or better accuracy to the individual emulators whilst requiring many fewer simulations, i.e., at a much-reduced computational cost.

**Algorithm 1:**
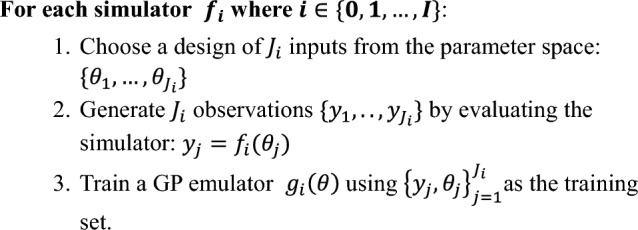
Individual Emulators for a cohort of size *I*

#### Latent-Feature Emulators

For our first method of cohort emulation, we seek to generate a single emulator for the entire cohort using latent features as additional training inputs. We assume that for each system, ${S}_{i}$, there is some set of latent parameters, ${l}_{i}\in \mathcal{L},$ which quantifies the difference between systems. In some cases, the choice of latent features may be clear, for example, if the real-world system being modelled was a chemical reaction in a set of differently sized pipes, then the latent features might be the length and diameter of each pipe. In other cases, the differences between each system instantiation may be less clear, and in this case it may be necessary to learn a latent-feature representation from additional information. For example, in our case studies where each system is a patient heart, we learn an encoding of cardiac anatomy from cardiac CT images using a reduced dimensional basis [11]. In other cases, perhaps where additional information is unavailable, we can use a purely empirical approach to find a latent representation ${l}_{i}$ characterizing the difference between each system. For example, we can use machine learning models to learn an appropriate latent representation such as Gaussian process latent variable models (GP-LVMs) [48] or variational auto-encoders [49] in cases where the cohort size is sufficiently large. The latent-feature representation can be treated as additional model parameters. For example, in our first case study the latent features parameterise each patient’s anatomical model, rather than the mathematical equations governing the simulation of cardiac function. But in other examples, we may find latent features that have no physical interpretation.

To train the latent-feature emulators we concatenate the simulation datasets from each cohort member: ${\left\{{f}_{i}\right\}}_{i=1}^{I}$, where $I$ is the size of the existing cohort, with the latent features for each cohort member ${\left\{{l}_{i}\right\}}_{i=1}^{I}$ to form a hybrid dataset: ${D}_{{l}_{i}}={\left\{\left({\theta }_{j};{l}_{i}\right),{f}_{i}\left({\theta }_{j}\right)\right\}}_{j=1}^{{J}_{i}}$. The full dataset is then constructed by concatenating all ${{D}_{l}}_{i}$:

3 $${D}_{l}={\left\{\left({\theta }_{j};{l}_{i}\right),{f}_{i}\left({\theta }_{j}\right)\right\}}_{j=1, i=1}^{{J}_{i}, I}$$

and the emulator is trained using ${D}_{l}$ (or some samples from ${D}_{l}$). This generates a single emulator for the entire cohort with the intention that any new cohort members can be emulated without any simulations of $f,$ as long as the latent variables are known or can be estimated. This process is described in Algorithm 2.

By generating a single emulator for the entire cohort, the process of emulation and prediction is streamlined for both new and existing cohort members and it allows the user to better interrogate how the latent features affect individual cohort member predictions. However, this approach requires that a sufficiently large cohort already be simulated so that latent features can be learnt (should this be needed) and the single latent-feature emulator trained. Note that the latent-feature emulator has the same computational structure as a standard GPE, but with an expanded input space to accommodate the latent-feature information.

**Algorithm 2:**
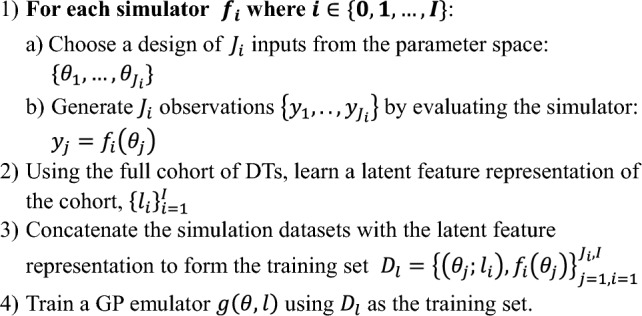
Latent feature emulator for a cohort of size *I*

#### Discrepancy Emulator

The second method we propose is to model the discrepancy between a new cohort member and the existing cohort. We assume that the simulator for the new cohort member can be described by a weighted sum of the previous members and some discrepancy term:

4 $$f_{I + 1} \left( \theta \right) = \mathop \sum \limits_{i = 1}^{I} a_{i} f_{i} \left( \theta \right) + \delta_{I + 1} \left( \theta \right),$$

where *a*_*i*_ are a set of weights that can be learnt or set by the user, and ${\delta }_{I+1}$ is the discrepancy model. We choose to consider $\delta$ as a function of $\theta$ and model it using a Gaussian process with mean ${\mu }_{{\delta }_{I+1}}\left(\cdot \right)$ and covariance $\kappa _{\delta _{I+1}}\left(\cdot ,\cdot \right)$.

We can then express the emulator for the new cohort member as a weighted sum of the emulators for the existing members:

5 $${g}_{I+1}\left(\theta \right)= \sum_{i=1}^{I}{a}_{i}{g}_{i}\left(\theta \right)+ {\delta }_{I+1} \left(\theta \right)$$

By the additivity property of Gaussian processes we know that ${g}_{I+1}(\theta )|\theta$ is normally distributed:

6 $${g}_{I+1}\left(\theta \right)|\theta \sim N\left(\sum_{i=1}^{I}{a}_{i}{\widehat{\mu }}_{i}\left(\theta \right)+{{\mu }_{\delta }}_{I+1}\left(\theta \right),\sum_{i=1}^{N}{a}_{i}^{2}{\widehat{\kappa }}_{i}\left(\theta ,\theta \right)+{\kappa }_{{\delta }_{I+1}}\left(\theta , \theta \right)\right)$$

and hence ${g}_{i}$ is a Gaussian process and we can learn ${\mu }_{{\delta }_{I+1}}$ and $\kappa_{\delta_{I+1}}$ using Gaussian process regression. Here, ${\widehat{\mu }}_{i}\left(\theta \right)$ and ${\widehat{\kappa }}_{i}\left(\theta ,\theta \right)$ are the posterior mean and covariance functions for ${g}_{i}$ conditional on the simulations used in their training. Note that as we are learning both the discrepancy term, ${\delta }_{I+1}$, and the weights, ${\left\{{a}_{i}\right\}}_{i=1}^{I}$, this introduces a structural non-identifiability. That is, for any ${\left\{{a}_{i}\right\}}_{i=1}^{I}$ it is always possible to learn a ${\delta }_{I+1}$ such that ${f}_{I+1}$ is well approximated. In practice, as interest only lies in prediction rather than inference of the parameters ${\left\{{a}_{i}\right\}}_{i=1}^{I}$ , this non-identifiability is immaterial – as long as the resulting emulator predicts well on unseen data we have a useful emulator. We can define the ${\left\{{a}_{i}\right\}}_{i=1}^{I}$ in multiple different ways (described below), but the underlying idea is that if we can learn ${\left\{{a}_{i}\right\}}_{i=1}^{I}$ in a way that reduces the discrepancy between a new cohort member and the existing cohort, then ${\delta }_{I+1}$ will require fewer training points to achieve the same predictive accuracy as an individual emulator.

We propose several methods for learning the weights ${\left\{{a}_{i}\right\}}_{i=1}^{I}$, including methods that learn the weights and discrepancy both sequentially and simultaneously. In the case where $I = 1$, i.e., the reference cohort contains only one anatomical model and the user is looking to incorporate a second model, the user might set ${a}_{1} = 1$, assuming that the cohort members are near identical, and then learn the discrepancy. In cases where $I> 1$, the weights can be treated as hyper-parameters and estimated during the GPE training. For example, we can augment the GP hyperparameters in the mean and covariance function with the ${\left\{{a}_{i}\right\}}_{i=1}^{I}$ and maximize the likelihood for all parameters simultaneously using a gradient-based optimizer. This method usually results in an emulator that uses all existing members of the cohort.

To create a sparser representation, we use a form of lasso regularization [50] to select a subset of the cohort, $J\subset \{1, \dots , I\}$, and set the remaining $\left\{{a}_{j}:j\notin J\right\}=0$. The non-zero weights, $\left\{{a}_{j}:j\in J\right\}$, can either be fixed to the values learned during the lasso regression, or again set as hyperparameters and learned during training. Essentially, we use a ${L}_{1}$ (lasso) regularization to select a sparse set of reference emulators, rather than using the entire cohort as reference. More specifically, we learn ${\left\{{a}_{i}\right\}}_{i=1}^{I}$ by minimising a regularized difference between the simulation of cohort member *I* + 1 and the weighted sum of the preceding *I* emulators evaluated at some set of inputs ${\theta }_{1},\dots ,{\theta }_{m}$, i.e.,

7 $$\widehat{a}=\underset{a}{\mathrm{argmin}}\sum_{j=1}^{m}{\left({f}_{I+1}\left({\theta }_{j}\right)-\sum_{i=1}^{I}{a}_{i}{g}_{i}({\theta }_{j})\right)}^{2}+\lambda {\Vert a\Vert }_{1},$$

where *λ* is a regularization parameter that can be selected by cross-validation, with larger values inducing more sparsity (i.e., causing the set $J$ to be smaller). Note that this minimisation problem requires no further simulations from the first $I$ geometries, only the $m$ simulations from the $I+1$ th geometry. The total computational complexity of solving this minimisation problem via lasso regression and subsequently training a discrepancy emulator is $\mathcal{O}({I}^{3}+{I}^{2}m+{m}^{3})$, whereas the computational complexity of simply training an individual emulator is $\mathcal{O}\left({m}^{3}\right)$. In our examples, the cost of solving this optimisation problem and subsequently training an emulator is always lower than doing additional simulation. The step-by-step method for training a discrepancy emulator is described in Algorithm 3.

Discrepancy emulators have the advantage of learning directly from the existing cohort, do not require any latent variable representation, and allow a user to add cohort members sequentially (potentially as data are gathered). However, they still require at least one fully trained emulator as an initial reference and in cases where cohort members differ significantly, there is the possibility that we gain no advantage by attempting to transfer knowledge between cohort members.

**Algorithm 3:**
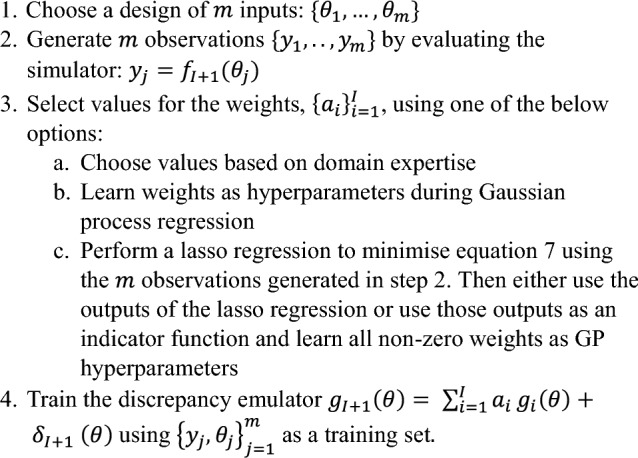
Training a discrepancy emulator with an existing cohort of size *I*

#### Kernel Interpretation

We can interpret GP regression methods as kernel methods [51] in order to understand the hypothesis space of functions considered by each of the three methods. In general, once we choose a kernel $\kappa$ in GP regression, we have specified a space of functions in which we believe ${f}_{i}(\theta )$ lies. This space is the closure of the set of functions span $\left\{\kappa \left(\theta , \cdot \right):\theta \in \Theta \right\}$, i.e., all linear combinations of $\kappa \left(\theta , \cdot \right)$ plus limit points. This is the reproducing kernel Hilbert space (RKHS) associated with $\kappa$, which we will denote as ${H}_{\kappa }$. For the individual emulator approach, we select a $\kappa$ in each case independently.

The latent and discrepancy emulators can then be viewed as extending or enriching ${H}_{\kappa }$ in two different ways. For the latent emulator, we use product kernels on the Cartesian product of the space of possible $\theta$ values, $\Theta ,$ and the space of latent features $\mathcal{L}$. If $\left(\theta , l\right)$ and $\left(\theta^\prime, l^{\prime}\right)$ are two elements of $\Theta \times \mathcal{L}$, then the product kernel is

$$K\left(\left(\theta , l\right), \left({\theta }^{\prime}, {l}^{\prime}\right)\right)= \kappa \left(\theta , {\theta }^{\prime}\right){\kappa }_{\mathcal{L}}(l, {l}^{\prime}),$$

where ${\kappa }_{\mathcal{L}}$ is the kernel chosen on $\mathcal{L}$. This makes the space of underlying functions modelled by the latent emulator the tensor product of ${H}_{\kappa }$ and ${H}_{{L}_{\kappa }}$. This model assumes that each ${f}_{i}$ lies in the same Hilbert space, ${H}_{\kappa },$ and we index across these individual functions using latent information $l$. In contrast, for the discrepancy emulators we are adding basis vectors corresponding to the previously learnt emulators. In other words, we are considering the closure of the set span $\left\{\kappa \left(\theta , \cdot \right) :\theta \in\Theta \right\}\cup \left\{{g}_{i}\left(\theta \right):i=1, \dots , I\right\}$ as our hypothesis space for ${f}_{I+1}$. These additional basis vectors can considerably enrich the space, and may simplify the task of learning ${\delta }_{I+1}$. It will not usually be possible *a priori* to say which approach will be more effective.

#### GPE Training and Validation

We use ${R}^{2}$ to evaluate the predictive accuracy of the emulators and the independent standard error (ISE) as a measure of the uncertainty quantification of each model. ${R}^{2}$ is a measure of the accuracy of the GPE prediction compared to the variance of the data:

8 $$R^{2} = 1 - \frac{RSS}{{TSS}},$$

where $RSS$ is the residual sum of squares (proportional to the mean square prediction error) and $TSS$ is the total sum of squares (proportional the variance of the output). An *R*^2^ close to 1 indicates high predictive accuracy; an *R*^2^ of zero indicates a predictive accuracy no better than always predicting the mean of the data. A negative ${R}^{2}$ indicates that the model prediction is worse than always predicting the mean of the data. In order to account for the uncertainty in our emulators we report an average *R*^2^ calculated using 1000 samples taken from the posterior distribution of the GP emulator.

The ISE counts the number of data points that fall within two standard deviations of the posterior mean prediction, and is reported as a percentage. In other words, it measures the empirical coverage of the GPE predictive distribution. Because the distributions are Gaussian, we ideally want the ISE to be approximately 96%.

To train and validate the models, each cohort member’s simulation data are randomly divided into training and testing sets using an 80:20 split. In some cases, emulators are trained on subsets of the training data (i.e., to test the effect of the number of training points on predictive accuracy). In such cases, we sample from the training set 5 times to generate these subsets, train an emulator on each subset, and average the ${R}^{2}$ and ISE scores over the 5 replicates. Reported ${R}^{2}$ and ISE values are always computed on the test set (i.e., on unseen data).

### Case Studies

#### Whole-Heart Electrophysiology

As our first case study, we used the cohort of 19 cardiac patients presented in [11]. The imaging data were collected as part of a prospective study approved by the Health Research Authority of St Thomas' Hospital, London, UK (18/LO/1803). The study conformed to the Declaration of Helsinki (reference ID 15/LO/1803) and all participants provided written, informed consent.

Each patient is represented by a mesh extracted from CT images, representing the geometry of their heart. In this case, candidate latent features were generated using a statistical shape model (SSM) trained solely on a cohort of mesh geometries. Meshes are represented as a linear combination of basis vectors (modes) chosen by a form of principle component analysis (PCA) [11] so that the first mode represents the direction of greatest variation between points, and so on. Because we need a finite low dimensional set of latent features, we truncate the latent representation and use just the first 9 modes, which capture more than 90% of the explained variance. The reaction-eikonal model of electrophysiology [52] is used to simulate activation times across the surface of the cardiac mesh. In the reaction-eikonal model, the main parameter that can be modified is the conduction velocity ($CV$) which is treated as homogeneous within each region of the heart. We used a transversely isotropic model, leading to one $CV$ in the fibre direction, and a different one transversely to the fibre direction. Similar to [12], the fibres in the ventricles were assigned using a rule-based method, setting the fibre direction from 60 degrees (endocardium) to − 60 degrees (epicardium). Atrial fibres were assigned using an atlas-based method mapping the fibres from an ex-vivo DTMRI. To model the fibre-transverse $CV$ we used an anisotropy ratio, $k$, so that $CV$ in the cross-fibre direction is $k \times CV$ . To model the fast activation that occurs naturally in the heart, we included a 1-element layer in the endocardium of the ventricles, simulating the Purkinje network [53]. In the atria we included a region comprising part of the left atrium and the right atrium to simulate the Bachmann bundle [54]. These two structures were modelled as isotropic materials. The CV was increased with respect to the myofibre CV by a factor of ${k}_{FEC}$ for the fast endocardial conduction layer and ${k}_{BB}$ for the Bachmann bundle. In total, six parameters were varied ($C{V}_{ventricles}, C{V}_{atria}, { k}_{ventricles}, {k}_{atria}, { k}_{FEC}, { k}_{BB}$). The activation times in each mesh were simulated at 180 different parameter combinations for each patient, sampled via latin hypercube sampling. The activation times were then summarised as the total activation times over the atria and ventricles, ${A}_{TAT}$ and ${V}_{TAT},$ i.e., the maximum activation time minus the minimum activation time over the spatial map of each chamber. ${A}_{TAT}$ and ${V}_{TAT}$ represent QRS and P-wave duration (measured from the ECG), respectively. Our ensemble of simulations thus contains 3420 observations, each with 15 input features (the 6 parameters of the reaction-eikonal model and the first 9 modes of the statistical shape model) and two outputs, ${A}_{TAT}$ and ${V}_{TAT}$. A representative case with the activation times from the EP simulations can be seen in Figure 8 in Appendix B.

#### Atrial Mechanics

As a second case study, we considered cardiac mechanical function using a cohort of 10 meshes, where each patient is represented by a unique computational mesh of the left atria (LA), estimated from CT images [61]. This study complied with the Declaration of Helsinki and the protocol was approved by the West Midlands Coventry and Warwick ethics committee and the London-Harrow ethics committee (clinical trial REC numbers 14/WM/1069 and 18/LO/0752) . The study was conducted in accordance with the local legislation and institutional requirements. Each patient provided written informed consent, and images were anonymised prior to analysis.

LA stiffness was estimated by fitting simulated output features of deformation to those derived from patient data. Healthy atrial function can be separated into an active and passive component. The passive filling and emptying of the LA was simulated with consideration of physiological constraints imposed by surrounding structures. We represented the LA myocardium using a transversely isotropic material law, which included a scaling parameter, $\alpha$, that scaled stiffness parameters along the fibre direction and in transverse and fibre-transverse planes, with the anisotropy ratio kept constant. We assumed that myocardial properties vary across the LA, therefore the LA was split into five regions (anterior, posterior, septum, lateral and roof) and each region was considered to have its own independent material properties (${\alpha }_{region}$). Passive atrial function is partly driven by an increasing chamber pressure as the LA fills. In the simulator, the initial chamber pressure was the end-diastolic pressure (EDP) and this pressure increases as the LA fills, reaching its peak pressure (the end-systolic pressure (ESP)) at the end-systolic time-point. LA deformation reaches its peak at the end-systolic time-point (ES). Physiologically, LA motion is constrained by the presence of a fibrous pericardium that restricts normal outward motion. The pericardium was modelled using normal springs applied to the LA epicardium, where pericardium spring stiffness varies spatially as a function of ${k}_{peri}$. The greatest constraint applied by the pericardium was on the LA roof and this decreased smoothly towards the LA mitral valve. The pericardium penalty threshold (PTH) represents the boundary beyond which no effect of the pericardium is applied to the LA epicardium. Nine parameters were varied in total (${\alpha }_{anterior},$ ${\alpha }_{posterior}, {\alpha }_{septum}, {\alpha }_{lateral}, {\alpha }_{roof}, EDP, ESV, {k}_{peri}, PTH$) and we simulated passive atrial deformation at 200 different parameter combinations. The output features describing the simulated deformation were LA volume (at ESV) along with global and regional displacements at ES. The displacements, ${d}_{global}, {d}_{anterior}, { d}_{posterior}, { d}_{septum}, { d}_{lateral}$ and ${d}_{roof}$, are calculated at ES by averaging the displacement of the mesh element centres over the whole mesh, anterior, posterior, septum, lateral and roof regions, respectively. It should be noted that we do not have a latent-feature representation of this case study, but are still able to test the discrepancy emulator method.

A representative case showing the regional definitions of the patient mesh (panel A) as well as the simulated outputs used for emulator training (panel B) are shown in Figure 9 in Appendix C.

## Results

### Whole-Heart Electrophysiology

#### Individual emulators

**Fig. 1:**
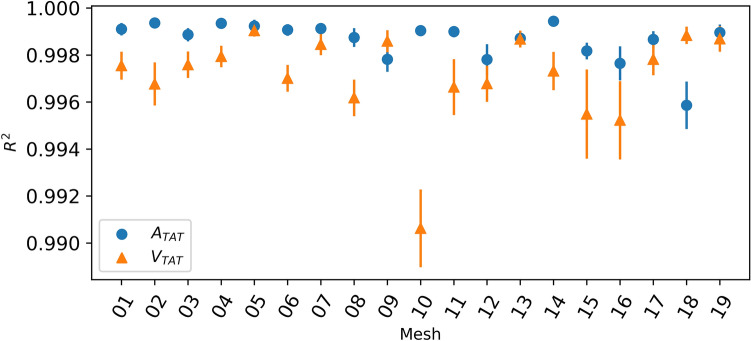
The predictive ${R}^{2}$ score over the test data of for each individual emulator trained with $m=$ 144 training points from simulations over each patient mesh. The error bars indicate ± 1 standard deviation over the 1000 samples from the GP posterior used to calculate the ${R}^{2}$

We trained 19 independent GPE ensembles, one for each patient, emulating both ${A}_{TAT}$ and ${V}_{TAT}$ independently for each patient. Each emulator was trained using 144 simulations of the cardiac model and tested using a further 36 simulations. To assess the predictive skill of the emulators, we sample from each GPE 1000 times and then calculate the mean and standard deviation of ${R}^{2}$, accounting for the uncertainty in the emulator. For every emulator the mean ${R}^{2}$ was greater than 0.990 for both ${A}_{TAT}$ and ${V}_{TAT}$ when using a training set of 144 simulations See Fig. 1.

We also tested the effect of reducing the number of training points. Table 1 shows the mean ${R}^{2}$ averaged over all 19 emulators as the number of training points increases. Even with a small number of training points, the predictive accuracy is relatively high, with ${R}^{2}>$ 0.990 for both ${A}_{TAT}$ and ${V}_{TAT}$ using only 60 training points. To contextualise these values, the average variance of ${A}_{TAT}$ over the test sets for each patient is 3110.8, therefore an ${R}^{2}$ of 0.895 corresponds to a mean squared error of approximately 330, whereas an ${R}^{2}$ of 0.999 corresponds to a mean squared error of approximately 3. The equivalent mean squared errors for ${V}_{TAT}$ are 70 and 0.7.

**Table 1:** Individual emulator mean ${R}^{2}$ scores with different training set size, $m$, averaged over the cohort of 19 meshes

$\mathrm{m}$	*20*	*40*	*60*	*80*	*100*	*120*	*140*
${A}_{TAT}$	0.895	0.988	0.995	0.996	0.998	0.998	0.999
${V}_{TAT}$	0.915	0.977	0.988	0.992	0.994	0.995	0.997

#### Latent-Feature Emulators

**Fig. 2:**
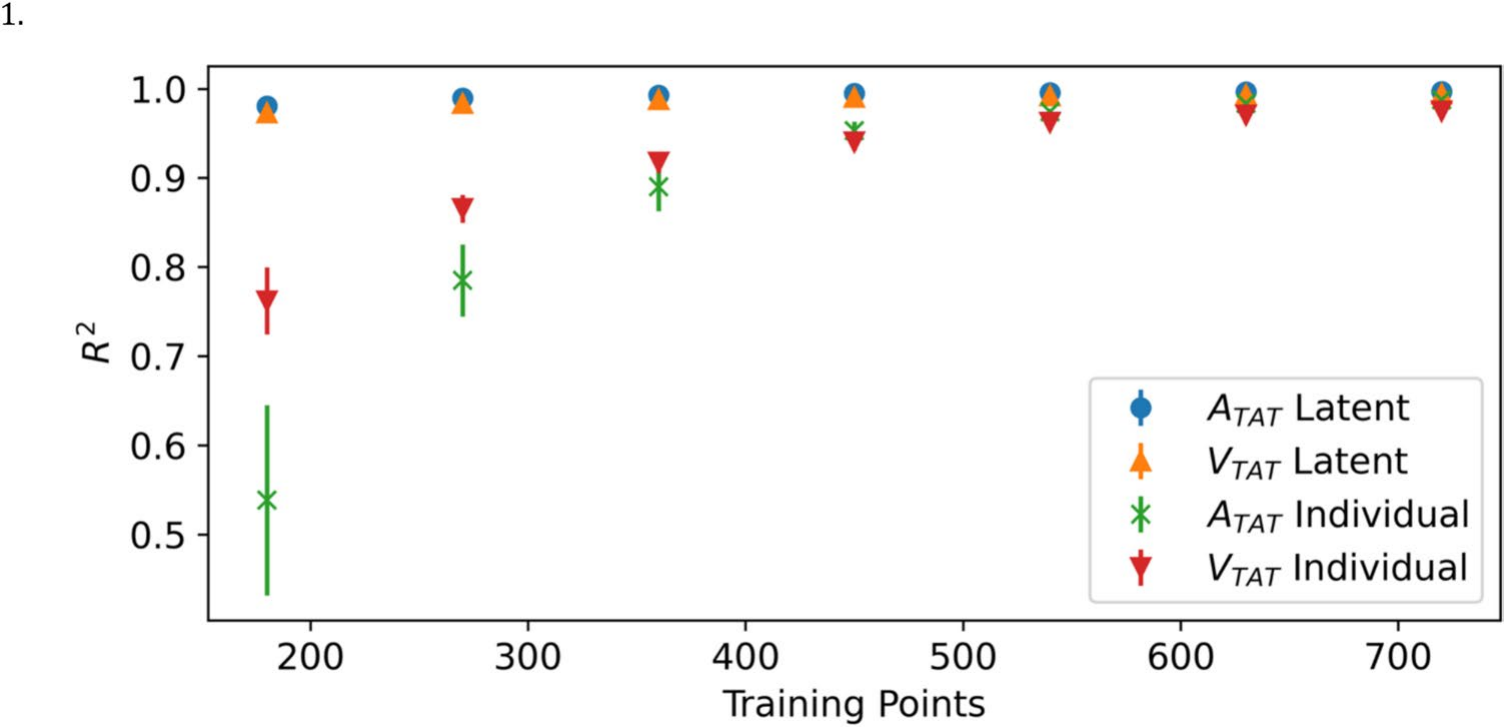
The average predictive *R*^2^ over the test data of the 19 latent-feature emulators (each one leaving out one mesh) evaluated on test data from the meshes used to train the emulators, compared to an ensemble of individual emulators trained using the same number of training points. The error bars indicate ± 1 standard deviation over repeated samples from the training set and the GP posterior

We trained latent-feature emulators using 18 of the 19 patient meshes, reserving the 19th mesh as test data, and then repeated this process for each mesh. Each emulator was tested on data from both the left-out mesh and the unseen test data from the 18 meshes used to train the emulator. Training sets were randomly selected from the total training data and this process was repeated 5 times. As a comparison, for each latent-feature emulator we trained a corresponding ensemble of 18 individual emulators using the same number of training points, i.e., if 180 training points from the cohort were used to train the latent-feature emulator, then each individual emulator was trained with 10 points sampled from the training data for that individual. This ensemble of individual emulators was then used to evaluate the test sets for the left-in and left-out meshes. For the left-in case each individual emulator was used to predict its own test data and for the left-out case each of the 18 emulators was used to predict the test data from the left-out patient. The ISE for all latent-feature models ranged between 94% and 96%, indicating that the latent-feature GPEs have well calibrated uncertainty. On the data from the 18 meshes (i.e., on meshes included in training but at unseen $\theta$ values), the emulator performs well, with ${R}^{2}>$ 0.950 for both outputs even at the lowest training set sizes (the equivalent of 10 training points per mesh), and it increases to $>$ 0.997 for ${A}_{TAT}$ and $>$ 0.994 for ${V}_{TAT}$ as the total training set size reaches 720, see Fig. 2. This is similar predictive accuracy to training individual emulators on 120 training points, but with the equivalent of 40 training points per emulator. The latent-feature emulator consistently outperforms the ensemble of individual emulators in low-data regimes, indicating that learning the additional latent-feature dependence of the outputs can lead to increased computational efficiency.

**Fig. 3:**
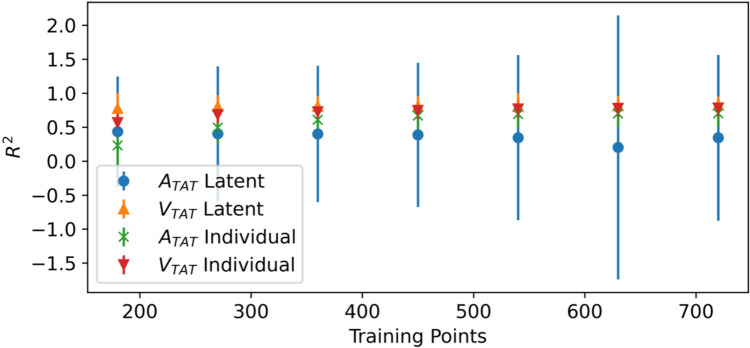
The average predictive *R*^2^ over the test data of the 19 latent-feature emulators (each one leaving out one mesh) evaluated on test data from the left-out meshes, compared to an ensemble of individual emulators trained using the same number of training points. The error bars indicate ± 1 standard deviation over repeated samples from the training set and the GP posterior

**Fig. 4:**
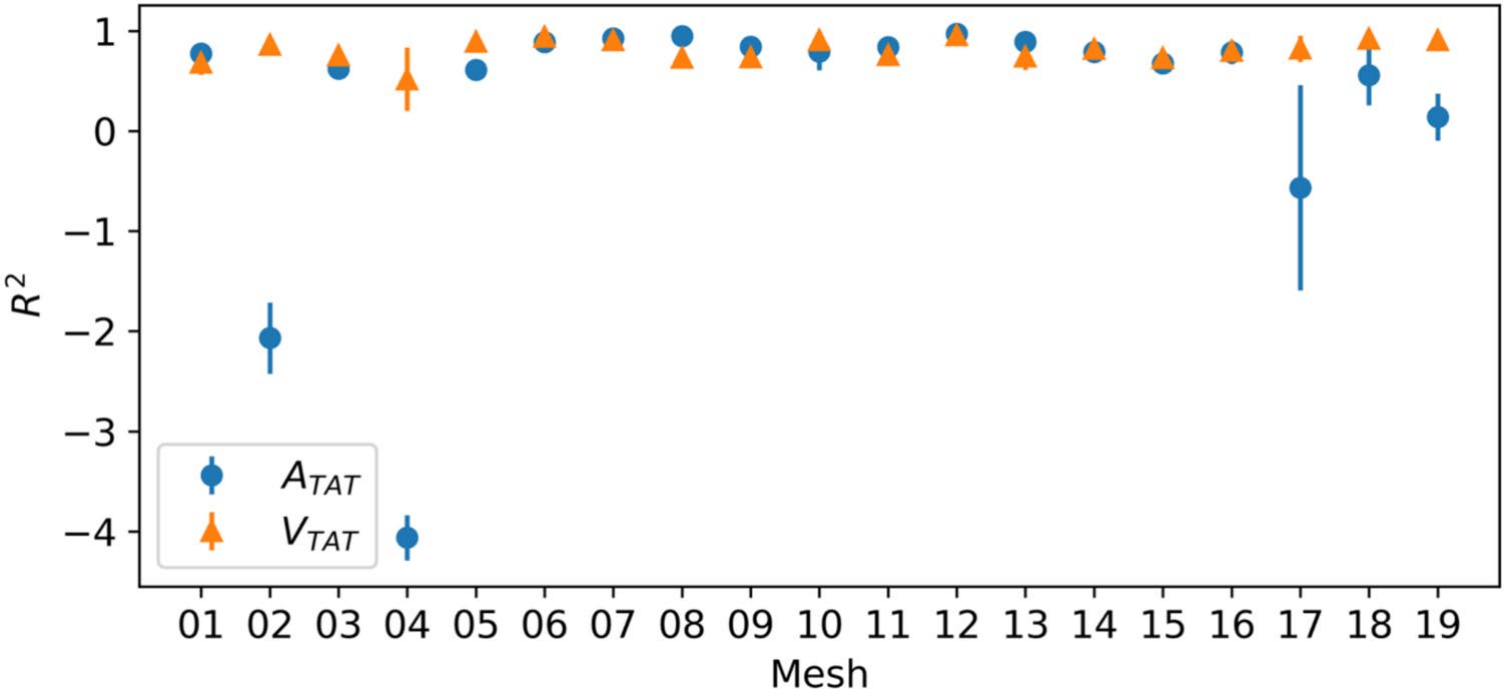
The predictive *R*^2^ over the test data of each of the 19 latent-feature emulators, trained with *m* = 720 training points, evaluated on test data from the left-out mesh. The x axis indicates which mesh was left-out during training. The error bars indicate ± 1 standard deviation of the *R*^2^ over repeated samples of the 720 data points and the GP posterior

We had hoped that by learning this latent-feature dependence we would be able to predict outputs for completely unseen meshes, as long as we knew their corresponding latent-feature representation. However, when testing on new patients (i.e., the left-out mesh), the ${R}^{2}$ is significantly lower, and never above 0*.*9 for either output, see Fig. 3. The results are similar for the ensemble of individual emulators, though they have lower uncertainty. This indicates that there is potentially some overfitting to the left-in meshes in the latent-feature emulator. This illustrates a potential problem with the latent-feature emulator approach if practitioners sought to use it to predict unseen meshes: patients that are very different to existing patients in the tapestry are difficult to predict as we extrapolate beyond the training set. Figure 4 shows the ${R}^{2}$ for each left-out mesh for each of the latent-feature emulators with $m =$ 720 training points. Here it is clear that for most of the left-out meshes, the predictive accuracy is relatively high, but the approach is inconsistent, with some unseen meshes very poorly predicted. As the number of patients in the tapestry increases, we would expect to see more consistent predictive accuracy for unseen meshes. Note that to determine whether or not a patient model has been well emulated by the latent-feature emulator requires some simulations from the full DT for the new patient.

#### Discrepancy Emulators

We compare six different variations of the discrepancy emulator (Eq. 4):

1. ${g}_{\delta } : a = 1$, discrepancy emulator with a single reference emulator and $a = 1$
2. ${g}_{\delta } : a = {a}_{r}$, discrepancy emulator with a single reference emulator and $a$ learned via least squares regression
3. ${g}_{\delta h} : a = {a}_{h}$, discrepancy emulator with a single reference emulator, and $a$ learned with the GPE hyperparameters
4. ${g}_{\delta c} : \{{a}_{i}\} = \{{a}_{ih}\},$ discrepancy emulator using the full cohort of reference emulators and $\{{a}_{i}\}$ learned with the GPE hyperparameters
5. ${g}_{\delta c} : \{{a}_{i}\} = \{{a}_{il}\},$ discrepancy emulator using the full cohort of reference emulators and $\{{a}_{i}\}$ learned using lasso regression (${L}_{1}$ regularisation).
6. ${g}_{\delta c} : \{{a}_{i}\} = \{{a}_{ind}\},$ discrepancy emulator using the full cohort of reference emulators. A subset $\mathrm{J}$ of the $\{{a}_{i}\}$ is selected as non-zero using lasso regression, and these are then estimated with the GP hyperparameters.

We compare these to an individual emulator trained on the same number of points, *g*_1_. We include methods 1, 2 and 3 to show the potential reduction in computational cost when using a single reference emulator, as this may be key for practitioners who are building a cohort from scratch or who have invested considerable computational resources into their first cohort member. In these cases reference emulators were selected randomly from the existing cohort (over 5 repetitions). In the cases where lasso regression is used, ${g}_{\delta c} : \{{a}_{i}\} = \{{a}_{il}\}$ and ${g}_{\delta c} : \{{a}_{i}\} = \{{a}_{ind}\}$, we seek to solve the minimisation problem in Eq. 7. The ${L}_{1}$ penalty induces sparsity in the estimates $\{{a}_{i}\}$ reducing the number of emulators used as references. The regularisation hyperparameter, $\lambda$, was chosen via cross-validation. As our references we use the emulators trained with 144 training points (the maximum available from the training set).

**Fig. 5:**
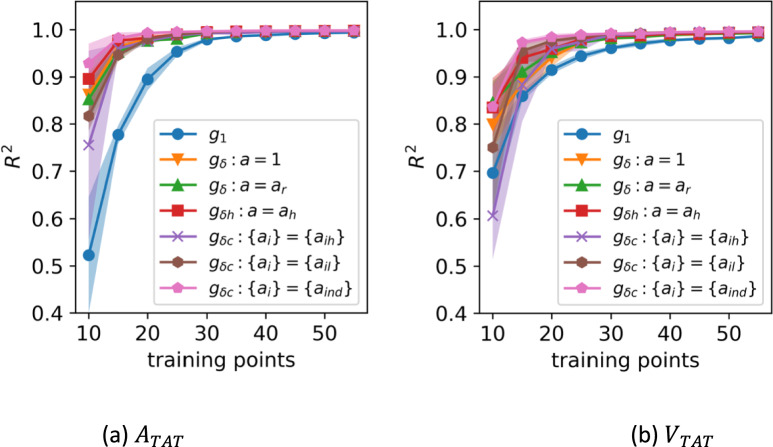
${R}^{2}$ over the test data for the six discrepancy methods compared with an individual emulator (${g}_{1}$) as the number of training points increases from 10 to 60 for ${A}_{TAT}$ and ${V}_{TAT}$. The shaded areas indicate ± 1 standard deviation over repeated samples from the training set and the GP posterior

**Fig. 6:**
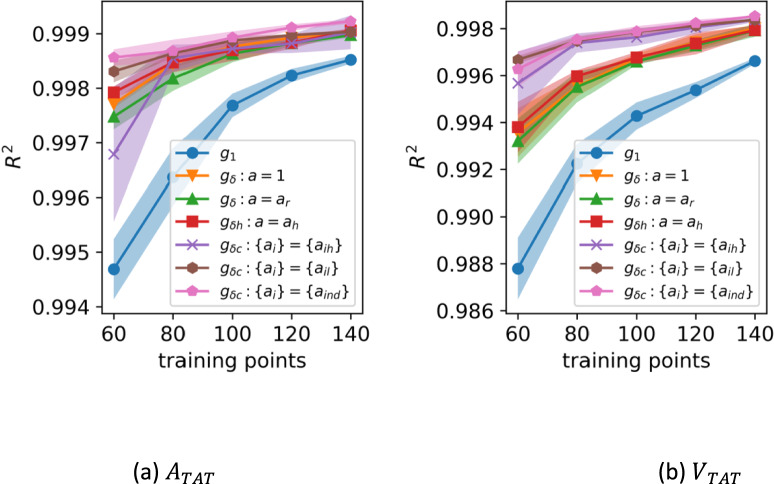
*R*^2^ over the test data for the six discrepancy methods compared with an individual emulator (${g}_{1}$) as the number of training points increases from 60 to 140 for ${A}_{TAT}$ and ${V}_{TAT}$. The shaded areas indicate ± 1 standard deviation over repeated samples from the training set and the GP posterior

Figures 5 and 6 show the varying *R*^2^ over the test data as the number of training points increases. For $m =$ 20 training points and above, each of the discrepancy methods has a higher ${R}^{2}$ than the individual emulator. Figure 5 shows the ${R}^{2}$ for $m$ between 10 and 60 and Fig. 6 shows the ${R}^{2}$ for $m$ between 60 and 140. These figures have been split to increase the legibility at higher values of $m$.

As the training set size increases, the discrepancy methods continue to outperform the individual emulators, though the difference in performance becomes negligible. Table 2 shows the same ${R}^{2}$ scores for both ${A}_{TAT}$ and ${V}_{TAT}$ emulators as in Figs. 5 and 6 at $m =$ 20, 40 and 60, to more clearly illustrate the difference in predictive quality between the methods. For readability the uncertain quantification has been omitted from the table, but is the same as in the figures. The ISE for all models ranged between 93 and 97%, indicating that the GPEs are well calibrated.

**Table 2:** The mean predictive ${R}^{2}$ score for each of the discrepancy models for $m =$ 20*,* 40*,* 60

	$m=20$		$m=40$		$m=60$	
**Model**	${A}_{TAT}$	${V}_{TAT}$	${A}_{TAT}$	${V}_{TAT}$	${A}_{TAT}$	${V}_{TAT}$
${g}_{1}$	0.895	0.915	0.988	0.977	0.995	0.988
${g}_{\delta }:a=1$	0.980	0.941	0.996	0.988	0.998	0.994
${g}_{\delta }:a={a}_{r}$	0.976	0.952	0.996	0.989	0.997	0.993
${g}_{\delta h}:a={a}_{h}$	0.983	0.959	0.996	0.991	0.998	0.994
${g}_{\delta c}:\left\{{a}_{i}\right\}=\{{a}_{ih}\}$	0.976	0.959	0.995	0.994	0.997	0.996
${g}_{\delta c}:\left\{{a}_{i}\right\}=\{{a}_{il}\}$	0.980	0.976	0.997	0.994	0.998	**0.997**
${g}_{\delta c}:\left\{{a}_{i}\right\}=\{{a}_{ind}\}$	**0.993**	**0.984**	**0.998**	**0.994**	**0.999**	0.996

Though all of the discrepancy methods perform better than the individual emulators for all $m$, the best performing discrepancy emulators are those that incorporate the full cohort and use lasso regularisation to create a sparse set of reference emulators. The lasso selects the best subset of reference emulators for the new cohort member, with no extraneous or conflicting information coming from less similar patients.

### Atrial Mechanics

As we do not have a latent variable representation of the atrial stiffness dataset due to the small cohort size, we can only compare the individual emulator and discrepancy emulator approaches.

#### Individual Emulators

Table 3 shows the individual emulator ${R}^{2}$ for the 7 outputs in the atrial stiffness dataset for varying training set sizes, $m$, averaged over the full cohort. The atrial stiffness dataset is a more challenging emulation task than the electrophysiology data, with the emulators for ${d}_{posterior}$ and ${d}_{roof}$ unable to achieve an ${R}^{2}>$ 0.8 at $m =$ 140.

**Table 3:** The mean predictive ${R}^{2}$ over the test data for each of the seven outputs in the atrial stiffness case study for an individual emulator as the number of training points, $m$, increases

$\mathrm{m}$	${{\boldsymbol{d}}}_{{\boldsymbol{g}}{\boldsymbol{l}}{\boldsymbol{o}}{\boldsymbol{b}}{\boldsymbol{a}}{\boldsymbol{l}}}$	${{\boldsymbol{d}}}_{{\boldsymbol{a}}{\boldsymbol{n}}{\boldsymbol{t}}{\boldsymbol{e}}{\boldsymbol{r}}{\boldsymbol{i}}{\boldsymbol{o}}{\boldsymbol{r}}}$	${{\boldsymbol{d}}}_{{\boldsymbol{p}}{\boldsymbol{o}}{\boldsymbol{s}}{\boldsymbol{t}}{\boldsymbol{e}}{\boldsymbol{r}}{\boldsymbol{i}}{\boldsymbol{o}}{\boldsymbol{r}}}$	${{\boldsymbol{d}}}_{{\boldsymbol{s}}{\boldsymbol{e}}{\boldsymbol{p}}{\boldsymbol{t}}{\boldsymbol{u}}{\boldsymbol{m}}}$	${{\boldsymbol{d}}}_{{\boldsymbol{l}}{\boldsymbol{a}}{\boldsymbol{t}}{\boldsymbol{e}}{\boldsymbol{r}}{\boldsymbol{a}}{\boldsymbol{l}}}$	${{\boldsymbol{d}}}_{{\boldsymbol{r}}{\boldsymbol{o}}{\boldsymbol{o}}{\boldsymbol{f}}}$	${\boldsymbol{E}}{\boldsymbol{S}}{\boldsymbol{V}}$
20	0.365	0.316	0.076	0.307	0.126	0.220	0.733
40	0.674	0.680	0.480	0.703	0.542	0.529	0.855
60	0.782	0.789	0.614	0.809	0.660	0.650	0.900
80	0.813	0.820	0.677	0.837	0.711	0.687	0.911
100	0.837	0.828	0.718	0.867	0.751	0.711	0.924
120	0.853	0.851	0.738	0.889	0.777	0.748	0.935
140	0.865	0.864	0.767	0.897	0.800	0.762	0.939

#### Discrepancy Emulators

**Fig. 7:**
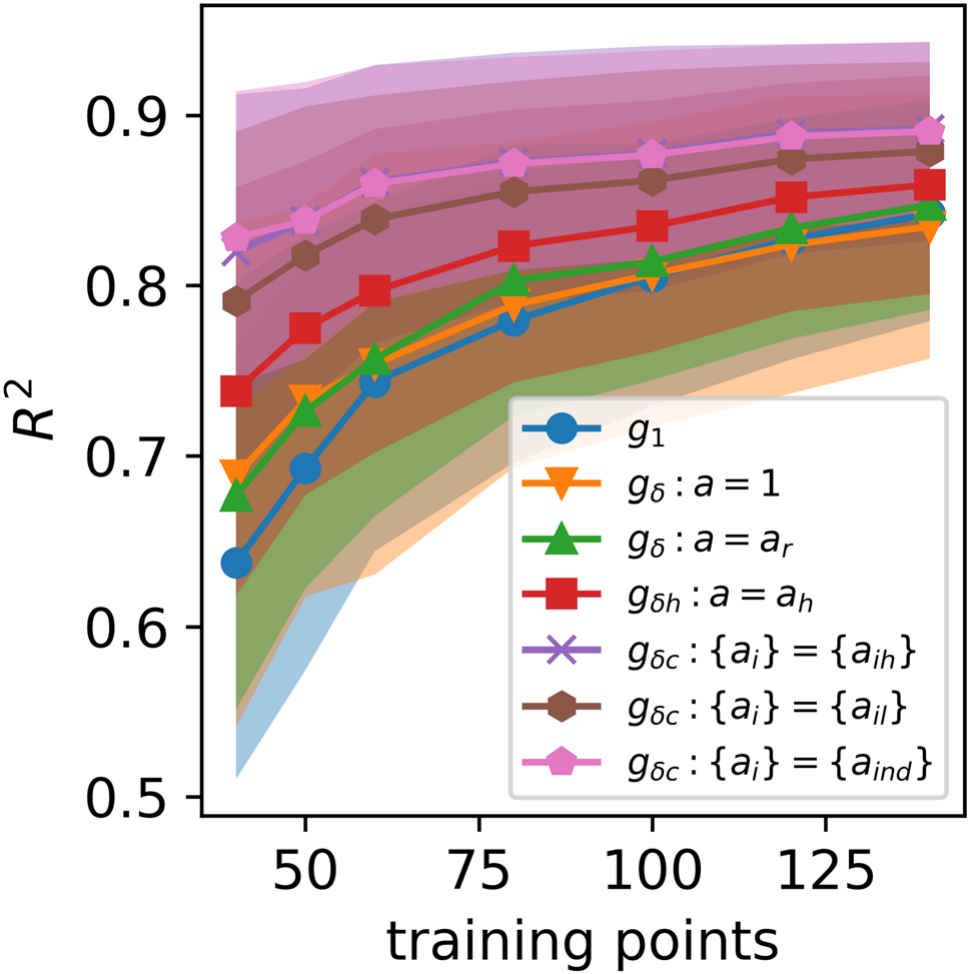
The predictive ${R}^{2}$ over the test data for each of the discrepancy methods averaged over the 7 outputs of the atrial stiffness model. The shaded areas indicate ± 1 standard deviation of the ${R}^{2}$ over repeated samples from the training set and the GP posterior

Figure 7 shows the predictive ${R}^{2}$ for each of the six discrepancy models compared to the individual emulators, averaged over the seven outputs in the atrial stiffness case study. In this case, individual emulators outperform the ${g}_{\delta } : a = 1$ model for $m>$ 120, but have a lower predictive accuracy than the other 5 discrepancy approaches. The full cohort discrepancy models, ${g}_{\delta c} : \{{a}_{i}\} = \{{a}_{il}\}$ and ${g}_{\delta c} : \{{a}_{i}\} = \{{a}_{ind}\}$, have similar predictive accuracy and the highest performance, as in the electrophysiology case. The ISE for all models ranged between 92% and 98%, indicating that the models are well calibrated. Table 4 gives ${R}^{2}$ for the ${g}_{\delta c} : \{{a}_{i}\} = \{{a}_{ind}\}$ discrepancy emulator, where lasso regression is used to select a subset of the ${a}_{i}$ to be non-zero.

**Table 4:** The predictive *R*^2^ over the test data for each of the seven outputs in the atrial stiffness case study for the ${g}_{\delta c} : \{{a}_{i}\} = \{{a}_{ind}\}$ (option 6) emulator as the number of training points, *m*, increases.

$\mathrm{m}$	${{\boldsymbol{d}}}_{{\boldsymbol{g}}{\boldsymbol{l}}{\boldsymbol{o}}{\boldsymbol{b}}{\boldsymbol{a}}{\boldsymbol{l}}}$	${{\boldsymbol{d}}}_{{\boldsymbol{a}}{\boldsymbol{n}}{\boldsymbol{t}}{\boldsymbol{e}}{\boldsymbol{r}}{\boldsymbol{i}}{\boldsymbol{o}}{\boldsymbol{r}}}$	${{\boldsymbol{d}}}_{{\boldsymbol{p}}{\boldsymbol{o}}{\boldsymbol{s}}{\boldsymbol{t}}{\boldsymbol{e}}{\boldsymbol{r}}{\boldsymbol{i}}{\boldsymbol{o}}{\boldsymbol{r}}}$	${{\boldsymbol{d}}}_{{\boldsymbol{s}}{\boldsymbol{e}}{\boldsymbol{p}}{\boldsymbol{t}}{\boldsymbol{u}}{\boldsymbol{m}}}$	${{\boldsymbol{d}}}_{{\boldsymbol{l}}{\boldsymbol{a}}{\boldsymbol{t}}{\boldsymbol{e}}{\boldsymbol{r}}{\boldsymbol{a}}{\boldsymbol{l}}}$	${{\boldsymbol{d}}}_{{\boldsymbol{r}}{\boldsymbol{o}}{\boldsymbol{o}}{\boldsymbol{f}}}$	${\boldsymbol{E}}{\boldsymbol{S}}{\boldsymbol{V}}$
20	0.737	0.718	0.621	0.803	0.590	0.593	0.919
40	0.875	0.860	0.734	0.893	0.742	0.741	0.952
60	0.894	0.888	0.777	0.912	0.795	0.788	0.963
80	0.899	0.901	0.801	0.922	0.807	0.808	0.963
100	0.909	0.902	0.802	0.929	0.817	0.813	0.964
120	0.917	0.906	0.817	0.933	0.839	0.833	0.967
140	0.917	0.909	0.820	0.938	0.844	0.838	0.967

## Discussion

We have presented two methods for the emulation of cohorts of digital twins using the transfer of information between individual cohort member meshes. The aim is to reduce the number of simulations required to generate an emulator for a new member of the cohort. The latent-feature emulator approach utilises latent variables that describe the difference between cohort members to train a single emulator for the entire cohort. The discrepancy emulator models the discrepancy between the existing digital twin cohort and a new member. As a baseline for comparison, we used individual emulators for each cohort member, and we tested our methods on two cohorts of cardiac models.

Though both methods aim to reduce the number of training points required to train an emulator, in practice they are solving slightly different problems. In the latent-feature emulator we ask whether we can approximate the entire cohort of emulators with a reduced training set per cohort member by learning an additional relationship between the simulator outputs and a learned latent representation. If $m$ training points are simulated for each of the $I$ cohort members, the computational complexity of training this model is $\mathcal{O}({\left(Im\right)}^{3})$ plus the cost of learning the latent representation. If the latent-feature emulator outperforms individual emulators for a given $m$ and the cost of learning the latent features is lower than the cost of generating new training points (which in our examples it is) then the latent-feature emulator is a more efficient use of computational resource.

For the discrepancy emulator we ask: if we have $I$ well trained emulators, and we want to add an $I+1th$ cohort member, are we better off independently training a new emulator or training a discrepancy emulator? If we have generated $m$ training points for the new cohort member, the cost of training a new emulator is $\mathcal{O}({m}^{3})$ and the cost of training the discrepancy emulator is $\mathcal{O}({m}^{3})$ plus the cost of learning the weights, ${\left\{{a}_{i}\right\}}_{i=1}^{I}$. If, for a given $m$, the discrepancy emulator has better performance than the new individual emulator, we propose that it is worth training a discrepancy emulator, because we assume that the cost of learning ${\left\{{a}_{i}\right\}}_{i=1}^{I}$ is significantly lower than generating more training points (i.e., doing more simulation).

The goal of cardiac digital twins (CDT) is to provide clinicians with a tool to aid treatment. For example, in atrial fibrillation the CDT could be used to simulate electrical activation patterns in order to guide catheter ablation therapy. Cardiac simulators are typically too computationally expensive to be practical tools, and although the community uses emulators to reduce this cost, at present the cost of building emulators is still prohibitive as each new patient necessitates a new set of simulations. Cohort methods aim to reduce the cost of training emulators in the setting where we have a stream of patients.

Another application of these methods is in the development of virtual patient cohorts. Virtual patient cohorts are sets of patient-specific medical mathematical models which may contain both real and synthetic patients [16]. Here, digital twins of real patients in the virtual cohort are generated by taking patient-specific measurements and mapping from the patient to the DT, and synthetic patients are generated by sampling from distributions over the population or generated by combining existing anatomical structures in the cohort to create so called virtual or digital chimeras [55, 56]. These virtual cohorts can then be used for in-silico trials [16, 57–60], to test outcomes over large groups of patients where in-vivo trials would be unfeasible. Cohort emulation methods can assist in the development of virtual cohorts by reducing the computational cost of simulating elements of the DT. For example, if an end user had a virtual cohort of cardiac meshes and wanted to simulate some model over each mesh, instead of running the simulator many times for each individual, they could use cohort emulation methods to reduce the number of required simulations.

### Electrophysiology

The aim of the two tapestry cohort methods is to reduce the number of training points required to achieve high predictive accuracy. With latent-feature emulators, it was possible to achieve similar predictive accuracy with the equivalent of 40 points per mesh (with 18 meshes in the tapestry) to individual emulators trained with $m$=120. We had hoped that the latent-feature emulator would allow us to accurately predict future cohort members with no further training. However, evaluating the emulator on left-out meshes resulted in reduced predictive capacity and a degree of inconsistency. Some unseen meshes were predicted well (${R}^{2}>$ 0.9) but some meshes had negative ${R}^{2}$ values, indicating catastrophic predictive performance. In cases with simpler latent spaces (or larger training sets with a larger number of meshes), the latent -feature emulator approach may be able to accurately predict unseen cases, our case study simply illustrates the potential problem of using such an approach.

In the electrophysiology example, we found that each version of the discrepancy emulator had a higher predictive accuracy than a single emulator trained on the same number of points (for $>$ 20 training points), and that the full cohort emulators perform better than using a single reference emulator in the discrepancy model. The choice of reference emulators is important, as demonstrated by the fact that the methods that used a lasso regularisation to select the best references had the best predictive performance. The cohort discrepancy emulator using lasso regression as an indicator function, ${g}_{\delta c} : \{{a}_{i}\} = \{{a}_{ind}\}$, trained on 40 training points, performs as well as the individual emulator for ${A}_{TAT}$ and ${V}_{TAT}$ trained with 100 points. With 60 training points, cohort discrepancy emulators have an ${R}^{2}$ score for ${A}_{TAT}$ and ${V}_{TAT}$ equivalent to the individual emulator trained with 140 points. In general, the full cohort discrepancy emulators require fewer than half of the simulations to match the accuracy of an individual emulator.

### Atrial Mechanics

The atrial stiffness case study posed a more difficult emulation problem. Individual emulators trained using *m* = 140 training points could not achieve ${R}^{2}>$ 0.8 for two of the outputs. Two of the full cohort discrepancy methods, ${g}_{\delta c} : \{{a}_{i}\} = \{{a}_{il}\}$ and ${g}_{\delta c} : \{{a}_{i}\} = \{{a}_{ind}\}$ achieved ${R}^{2}>$ 0.8 for all outputs at $m =$ 120. The model ${g}_{\delta c} : \{{a}_{i}\} = \{{a}_{ind}\}$ outperformed the individual emulators (for all choices of $m$) when trained with $m =$ 80 and outperforms the individual emulators for 6 out of 7 outputs at $m =$ 60. As in the electrophysiology case, the discrepancy emulation approach can achieve the same predictive accuracy at approximately half the computational cost of adding a new cohort member.

### Limitations

Though both the latent-feature and discrepancy emulator approaches are capable of high predictive accuracy with small training sets compared to individual emulators, both have limitations. A key consideration when implementing the latent-feature method is how to learn a suitable latent-feature representation. In this paper we have used a method based on the cohort of geometries, but one might instead wish to learn latent features based on model outputs using a method such as the GP-LVM [48], which would require either a large number of simulations, or training individual emulators in order to generate a surrogate dataset on which to perform dimension reduction. Domain expertise can also be used when available. Learning a suitable representation may also be complicated by smaller cohort sizes. We have shown that the latent-feature approach has inconsistent predictive power on unseen geometries, but this can be improved by increasing the size of the initial training cohort, i.e., doing additional simulation.

The discrepancy emulator approach performed consistently well across all patient geometries and in both case studies. Even in the cases where we train a discrepancy emulator with a single reference emulator, it outperforms individual emulators trained on the same number of observations. However, this method relies on a degree of similarity between cohort members, and it is easy to imagine data generating functions where outputs vary wildly depending on the input parameters, as occurs, for example, in some oscillators. In such cases, the information learned from the cohort could make training the discrepancy emulator more difficult than simply learning an individual emulator. This problem is partially addressed by the lasso regularisation approach to selecting the non-zero weights, as with appropriate tuning of the regularisation variable, very dissimilar cohort members would likely be ruled out. The need to choose an appropriate set of weights, ${\left\{{a}_{i}\right\}}_{i=1}^{I}$, is another limitation as although these can be learned alongside the GP hyperparameters, it adds complexity and necessitates additional analysis for each new DT. Finally, note that although the method as stated here relies on an assumption of additive discrepancy, other functional forms (i.e., non-additive) can be used, possibly by transforming the outputs to return to an additive model. For example, if we believed the discrepancy was a multiplicative correction, then we can log transform the output, to regain an additive model.

We had originally hoped that the latent-feature method might let us emulate patients with only information about their cardiac geometries, that is, without having to run the simulator. Unfortunately, as we show in Fig. 4, the emulator had inconsistent performance on unseen geometries. This means that both methods still require simulator runs to add a new patient to the cohort. Though requiring fewer runs may reduce the computational bottleneck in some clinical settings, neither method presented here fully mitigates that bottleneck. This also introduces the problem of deciding how many simulations are required for each new patient. If an end user sought to minimise the total number of simulations required to reach some threshold of predictive accuracy, they could generate new simulations sequentially, testing predictive accuracy using a leave-one-out method, rather than splitting data into training and test sets.

### How Should I Weave my Tapestry?

In applications where a user has a cohort of DTs that they seek to emulate, there are various benefits to applying transfer or meta-learning methodologies. We have shown that it is possible to reduce the number of simulations required to train emulators, and also that it is possible to use methods such as lasso regression to learn subsets of the cohort that share similarities that can be leveraged to improve emulator quality.

That said, which method of cohort emulation one should use is dependent on various factors, including the number of existing simulations, computational budget, cohort size, ability to learn the latent features, and whether the cohort is already complete, or whether one expects to add new members. We believe that, depending on the use case, both methods may be useful to digital twin practitioners.

For example, if no computation has previously been performed, the cohort is complete, and we have features we believe will serve well as latent features, then the latent-feature emulator may be the best user choice. It produces a single, easily interpretable model, and we have shown in the electrophysiology case that it can provide comparable accuracy to individual emulators at significantly reduced simulation cost. However, we may have to re-train the model every time we add new cohort members, as we found that predictions on unseen geometries could be poor. Furthermore, it requires that the latent variables be known, or a model generated to learn them, such as a statistical shape model [11] or GP-LVM [48].

On the other hand, if one intends to add cohort members sequentially (i.e., as patient data are recorded) and there are simulations for a number of early cohort members, the cohort discrepancy method provides high accuracy with reduced computational costs without requiring a projection onto the latent space. The discrepancy emulator method could achieve equivalent predictive power to the individual emulators using half the simulation budget.

We hope that both methods presented here can be useful tools for practitioners seeking to leverage information from cohorts of digital twins.

## Supplementary Information

Below is the link to the electronic supplementary material.

Supplementary file1 (DOCX 5607 kb)

## Data Availability

The whole-heart electrophysiology simulation data are available from Zenodo (10.5281/zenodo.14639849) as is the atrial mechanics simulation data (10.5281/zenodo.14504006).

## Code Availability

Code for the discrepancy and latent-feature emulators is available at https://github.com/cwlanyon/GPyEm a Zenodo archive of the source code is available here: 10.5281/zenodo.15632953

## Materials Availability

Not applicable for this work.
