# Supplementary material for: Weaving the Digital Tapestry: Methods for Emulating Cohorts of Cardiac Digital Twins Using Gaussian Processes
Source: Ann Biomed Eng. 2025 Nov 17;54(2):514–30. doi: 10.1007/s10439-025-03890-0 (PMC12852307; doi:10.1007/s10439-025-03890-0)
Supplement: Supplementary file 1 — Supplementary file1 (DOCX 5607 kb) [file 10439_2025_3890_MOESM1_ESM.docx]

**Supplementary information.**

# Appendix A Gaussian process mean and covariance functions

For the Gaussian process emulators we used a linear mean function:

$\mu=\beta_{0}+\sum_{i=1}^{k} \beta_{i}\theta_{i}$ (A1)

We use a radial basis function (RBF) for the covariance function:

$\kappa\left( \theta,\theta' \right)=\sigma_{s}\exp\left( -\frac{1}{2}\left( \theta-\theta^{'} \right)^\top\Theta^{-2}(\theta-\theta^{'}) \right)$ (A2)

where $\sigma_{s}$is the output scale and $\Theta$ is a matrix of lengthscales. We estimate the hyperparameters using the ADAM stochastic gradient descent algorithm in PyTorch.

# Appendix B Cardiac Mesh Example


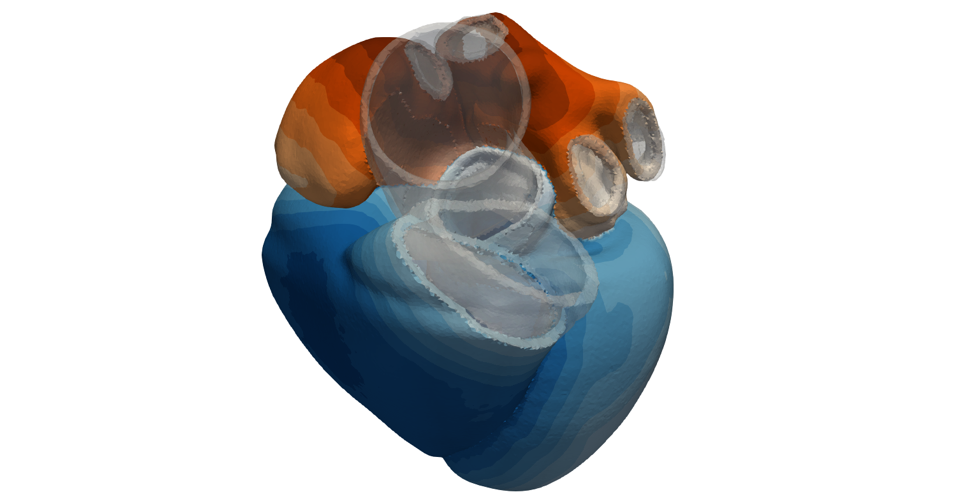


**Fig. 8**: Cardiac mesh for patient #02 from Rodero et al. [11]. Blue colours correspond to the ventricles, orange to the atria. Each segment represents a 10 ms increment, with darker colours representing earlier activation times.

# Appendix C Atrial mechanics representative case


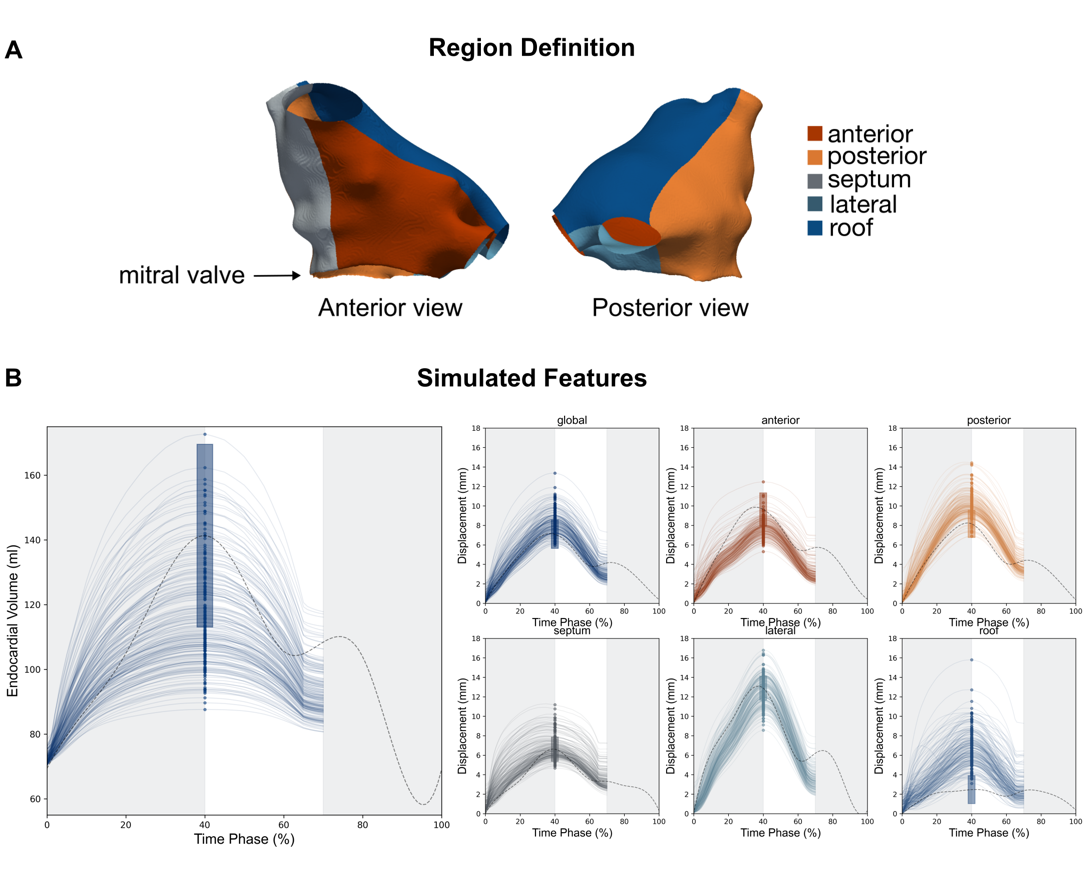


**Fig. 9**: Representative case from the atrial mechanics cohort. Panel A shows five regions on the representative LA mesh. Panel B shows simulated endocardial volume and surface displacements transients. The dots represent the volume and displacement values at ES, which we use for emulator training. The dotted line represents the patient-derived deformation.
